# A Highly Sensitive UPLC-MS/MS Method for the Quantification of the Organic Cation Transporters’ Mediated Metformin Uptake and Its Inhibition in Cells

**DOI:** 10.3390/molecules29215162

**Published:** 2024-10-31

**Authors:** Gzona Bajraktari-Sylejmani, Cindy Bay, Lukas Gebauer, Jürgen Burhenne, Johanna Weiss, Max Sauter

**Affiliations:** 1 Internal Medicine IX—Department of Clinical Pharmacology and Pharmacoepidemiology, Medical Clinic Heidelberg, Medical Faculty Heidelberg, University of Heidelberg, 69120 Heidelberg, Germany; cindy.bay@med.uni-heidelberg.de (C.B.); juergen.burhenne@med.uni-heidelberg.de (J.B.); max.sauter@med.uni-heidelberg.de (M.S.); 2 Institute of Clinical Pharmacology, University Medical Center Göttingen, 37075 Göttingen, Germany; lukas.gebauer@med.uni-goettingen.de

**Keywords:** HEK293, metformin, OCT, organic cation transporters, UPLC-MS/MS, verapamil

## Abstract

Metformin is the gold standard substrate for evaluating potential inhibitors of the organic cation transporters (OCTs). Here, we established a UPLC-MS/MS assay to quantify metformin in cell pellets with a range of 0.05–50 ng/mL using 6-deuterated metformin as an internal standard. We used an ion-pairing chromatographic approach with heptafluorobutyric acid, making use of a reverse-phase column, and overcame the associated ion-suppression via previously established post-column injection of aqueous ammonia. The assay was validated according to the Food and Drug Administration (FDA) and the European Medicines Agency (EMA) recommendations for bioanalytical methods. The established extraction procedure was simple, very fast and ensured almost 100% recovery of the analyte. The exceptionally sharp peak form and retention of the ion-pairing chromatography are superior to other methods and allow us to measure as sensitively as 0.05 ng/mL. We used the herein established and validated method to develop a cellular OCT inhibition assay by using metformin as a substrate and human embryonic kidney cells (HEK) overexpressing the OCTs 1-3. The method presented may be useful for identifying new OCT inhibitors, but also for drug–drug interactions and other pharmacokinetic studies, where accurate quantification of low metformin amounts in relevant tissues is mandatory.

## 1. Introduction

The organic cation transporters (OCTs) are important physiological membrane transporters with a central role in absorption, cellular accumulation, and renal and biliary excretion of drugs [1,2]. Therefore, testing the substrate and inhibitor capacity of new drugs for OCTs is suggested by regulatory bodies such as the Food and Drug Administration (FDA) and the European Medicines Agency (EMA). Metformin is the gold standard substrate used in in vitro testing of potential drug-drug interactions mediated by OCTs [3,4,5,6]. Most frequently, metformin is quantified using radiometric assays [7,8,9]. Even though radiometric assays are sensitive and selective, they require specially trained personnel and special infrastructure, including radioactive waste disposal, making them impractical and cost-intensive for many laboratories. Ultra-performance liquid chromatography coupled with tandem mass spectrometry (UPLC-MS/MS) methods is more practical without sacrificing the sensitivity and selectivity of radiometric methods. This has caused wide usage of these methods for the detection and quantification of drugs in different matrices. The majority of small molecule drugs have an amphiphilic nature and are therefore easily separated using reverse-phase chromatography and standard mobile phases. However, its biguanide structure [10] causes metformin to be highly hydrophilic, which causes difficulties in the chromatographic separation of this very small molecule. This is even more difficult when high sensitivity is required due to small sample amounts, e.g., in cell pellets of inhibition assays.

To achieve high sensitivity and good peak shape for highly hydrophilic substances, different chromatographic approaches are available, such as hydrophilic interaction chromatography (HILIC) or ion exchange chromatography. However, HILIC methods are usually employed to achieve a chromatographic separation of hydrophilic compounds, but this often affects the peak shape, which reduces the sensitivity of the method. More importantly, HILIC methods regularly suffer from decreased robustness, making their routine implementation challenging. Reverse-phase columns are therefore the gold standard for robust and reliable LC-MS/MS development. However, due to the high polarity of metformin, reverse-phase separations are challenging to develop, require hybrid columns, and often suffer from broad peak width and very little retention, resulting in low chromatographic resolution. Furthermore, most of the published assays so far were developed for plasma, where the concentrations are very high, thus not requiring sensitive measurements [11,12,13]. Recently, we developed a chromatographic separation of oligoarginine-lipid conjugates using trifluoroacetic acid (TFA) mobile phases with superior chromatographic characteristics compared to standard formic acid, which was combined with a post-column infusion strategy of aqueous ammonia to counteract the ion suppression caused by the TFA adducts [14]. The principle was anticipated to be used for the bioanalysis of arginine-related guanidino compounds with challenging chromatographic characteristics such as metformin. However, in our case, TFA did not achieve sufficient retention on standard C18 columns. Therefore, we performed the chromatography using heptafluorobutyric acid (HFBA) to achieve more hydrophobic ion pairing for optimal retention and used the same post-column infusion strategy with aqueous ammonia for adduct desolvation. Using HFBA, we obtained superior peak shape and width with very high retention compared to other chromatographic approaches and easily achieved our targeted lower limit of quantification of 0.05 ng/mL. The developed method was validated according to the pertinent guidelines of the FDA and EMA and was used to quantify metformin in cells. Using human embryonic epithelial cells (HEK) overexpressing the human OCT transporters 1-3 and metformin as a substrate, we established a model system for quantifying the OCT activity in cells and for testing compounds for their OCT inhibitory potential.

## 2. Results

### 2.1. Mass Spectrometry

**Figure 1:**
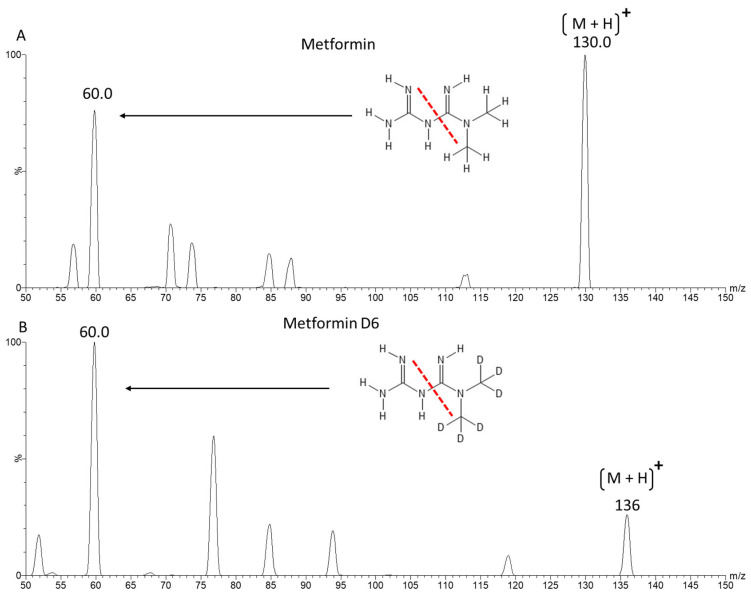
Product ion spectra of metformin (**A**) and metformin D6 (**B**) with multiple reaction monitoring (MRM). The precursor (M + H)^+^ and the most intense product ions used for quantification are labelled with their respective *m*/*z* values. The depicted chemical structure shows the cleavage site of the monitored mass transition with a red dotted line.

Using ESI positive ion mode, we achieved a very good ionisation of metformin and 6-deuterated metformin (metformin D6) with two dominant ion transitions resulting from the splitting of the biguanide molecule. For quantification, ion transitions *m*/*z* 130/60 were used for metformin and *m*/*z* 136/60 for metformin D6, as seen in Figure 1.

### 2.2. Method Development

**Figure 2:**
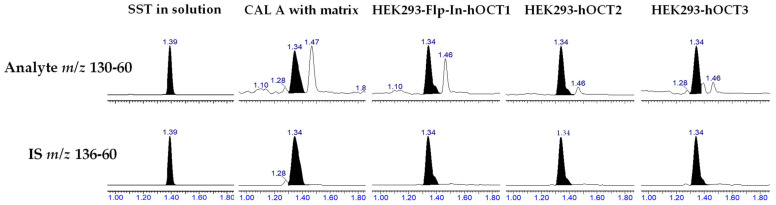
Chromatographs of metformin and the respective metformin D6 peaks, using HILIC chromatographic separation in a Waters Xevo TQXS. Shown are a system suitability test (SST), which was in solution, CAL A with blank matrix and real samples from the HEK293-Flp-in-hOCT1, HEK293-hOCT2 and HEK293-hOCT3 cell lines. The addition of the matrix negatively affected the peak shape and width achieved in solution. Furthermore, there were differences in peak shape among cell lines.

**Figure 3:**
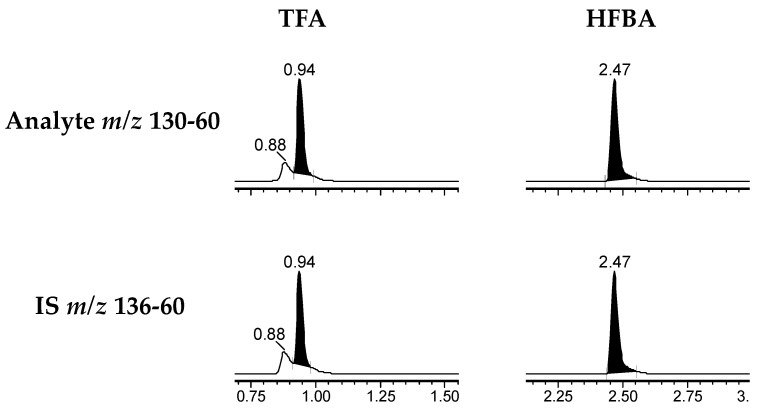
Chromatographic separation of metformin and D6-metformin in solution using the TFA and HFBA aqueous ammonia approaches in a Waters Xevo TQ-S with an Acquity BEH Peptide Column. Using TFA, the retention time was unsatisfactory, and a small shoulder peak can be seen. The change to HFBA caused an acceptable retention time as well as good peak shape and width.

**Figure 4:**
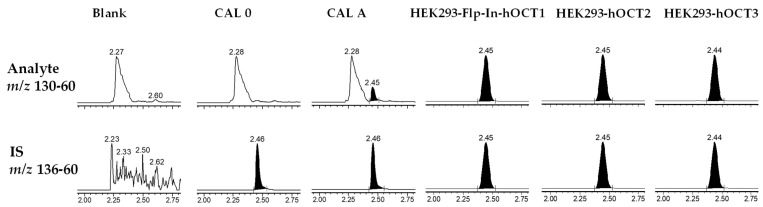
Chromatographs using HFBA separation on a Waters Acquity BEH Peptide Column in a Waters Xevo TQ-S. Shown are a blank matrix, CAL 0 (only IS) and CAL A, and real samples from the cell lines HEK293-Flp-in-hOCT1, HEK293-hOCT2 and HEK293-hOCT3.

Using HILIC chromatographic conditions on a Waters Cortecs HILIC column, we achieved a good chromatographic separation and a good peak shape of metformin in solution. However, when the matrix was added using protein depletion with acetonitrile, the peak shape was largely affected (Figure 2). Furthermore, different cell lines (HEK293-Flp-In-hOCT1, HEK293-hOCT2 and HEK293-hOCT3) had different peak shapes and small disturbing peaks (Figure 2). Therefore, we tested other approaches with a focus on chromatographic resolution and robustness. As these characteristics are usually achieved on reverse-phase columns, we tried a TFA-based methodology previously developed from our group that uses aqueous ammonia to resolve adducts of the perfluoro additives and avoid the normally associated ion suppression [14]. However, on a Waters Acquity BEH Peptide Column, the retention time achieved was unsatisfactory, as the peak eluted close to the column void volume and the peak had a small shoulder (Figure 3), indicating unsatisfactory chromatographic resolution and too little retention. Utilising the same principle, but instead of TFA, using the substantially more hydrophobic HFBA for ion-pairing chromatography, the peak shape in solution was excellent, with a width at baseline of 6 s. To establish a simple and rapid extraction compatible with the chromatographic methodology, we used protein precipitation of cell lysates using 7% HFBA, which efficiently depleted proteins from the samples and resulted in aqueous extracts that could be directly injected without further processing. Using the combination of this sample extraction methodology and the HFBA chromatography, interferences from the matrix were clearly separated (Figure 4), and no differences between the cell lines were observed. We thus proceeded with the validation of this HFBA-based assay.

### 2.3. Validation

Metformin and metformin D6 were very robust in our method, and all the QC samples (LLOQ, QC A, QC B and QC C) fulfilled the ±15% variation that is accepted for bioanalytical methods according to the FDA and EMA (Table 1). The absence of interfering signals in blank samples of all used cell lines demonstrated the specificity of the assay (Figure 4 for HEK293). Metformin was stable under all the tested conditions (Table 2). The recovery of the simple and rapid extraction procedure generating aqueous extracts was quantitative, demonstrating its feasibility for the highly hydrophilic metformin. Presumably due to the efficient depletion of proteins and lipids by precipitation with HFBA, the absolute matrix effects were negligible (Table 3). Additionally, the IS-normalised matrix effects were within the required ranges, proving the suitability of the deuterated IS.

### 2.4. Quantification of Metformin in HEK293-hOCT1-3 Cells: Uptake Kinetics and Inhibition

**Figure 5:**
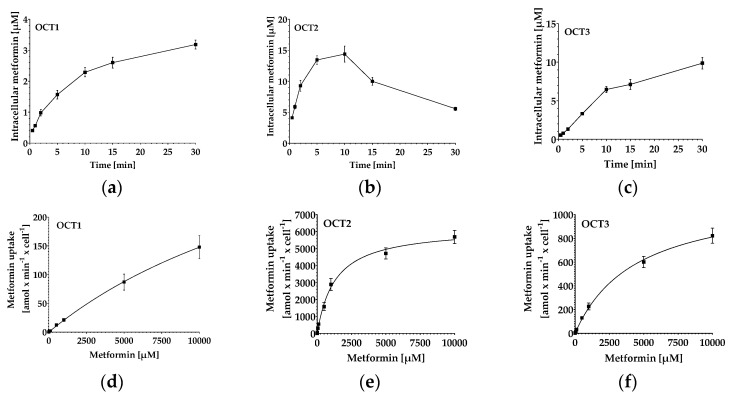
Uptake kinetics of metformin in OCT-overexpressing HEK293 cell lines. (**a**–**c**) Time-dependent uptake of metformin (20 µM for OCT1 and OCT3, 2 µM for OCT2). Data depict mean ± S.E.M. of n = 9–18. (**d**–**f**) Concentration-dependent uptake of metformin in OCT-overexpressing HEK293 cell lines. Cells were incubated for 5 min (OCT2 and OCT3) or 10 min (OCT1) with metformin. Data depict mean ± S.E.M. of n = 9.

After having established and validated the quantification method for metformin in cells, we tested its suitability by measuring the uptake kinetics of metformin in HEK293-Flp-In-hOCT1, HEK293-hOCT2 and HEK293-hOCT3 cells (Figure 5a–c). As expected, the metformin uptake followed Michaelis–Menten kinetics, with OCT2 being the OCT with the highest affinity for metformin and a greater velocity than OCT1 and OCT3 (Figure 5d–f, Table 4).

**Figure 6:**
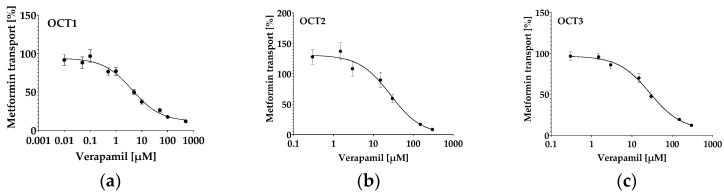
Inhibition of metformin transport in HEK293-overexpressing OCT1 (**a**), OCT2 (**b**) or OCT3 (**c**) by verapamil. Data depict mean ± S.E.M. of n = 9–21.

The well-known OCT1-3 inhibitor verapamil inhibited metformin uptake in all HEK293 cell lines overexpressing OCT1 (Figure 6a), OCT2 (Figure 6b) or OCT3 (Figure 6c), with IC_50_ values in the micromolar range (Table 4), whereas inhibition of OCT1 was most potent.

## 3. Discussion

Metformin is recommended by the FDA and EMA as the gold standard substrate for characterising inhibitor capacity of drugs for the OCT1-3 transporters [3,4,5,6]. This is plausible, considering that the small hydrophilic molecule metformin shows negligible passive diffusion through cellular membranes, and its transport through them relies entirely on transporter-mediated cellular uptake and efflux. Although there are numerous LC-MS/MS-based methods to measure metformin in plasma [11,12,13], in vitro cellular assays are generally conducted using radiometric assays [7,8,9]. Our goal was to establish in vitro screening models for the OCT1-3 inhibitor capacity of drugs by using HEK293 cells overexpressing these transporters. For this goal, we needed a very sensitive assay that can measure small amounts of metformin, allowing us to fully characterise strong inhibitors of the cellular uptake of metformin. As we screened the literature, it was clear that most of the methods to measure metformin are not suitable for this requirement, as they have very high LLOQs only suitable for measuring the high plasma concentrations [11,12,13]. Furthermore, in contrast to plasma, where one can use a sample volume of 100–500 µL, our cell pellets have an initial sample volume of only 0.5–2 µL. To the best of our knowledge, the only method published so far uses a custom-modified sample manager system and two LCs using a 384-well screening format, where all the sample preparation and injections are done in the same plate using a number of automated steps, which is, however, not available in every laboratory [15]. Although the method is highly applicable for a fast early screening approach, we wanted to quantify metformin very accurately. This seemed unlikely using a 384-well format without normalisation, as HEK293 cell lines usually do not grow confluent. Our assay is conducted in microtubes using an exact cell number (counted with our CASY® cell counter system) and using the exact cell volume (calculated by the CASY), which allows us to accurately calculate our sample volume for each experiment. The sample extraction method we used is very simple, with only one transfer step from the microtube to the 96-well measurement plate. Furthermore, because we conducted the protein precipitation with similar composition to our initial mobile phase conditions, no drying and reconstituting steps were needed. This not only saves time and consumables but also ensures minimal losses and variations throughout the sample preparation.

We aimed to achieve a robust method that allows for reliable separation of interferences and optimal sensitivity. This is especially necessary when using very small sample amounts such as in cell-based assays. Therefore, we required a chromatographic method with high retention to maximise resolution power and very sharp peaks for maximal sensitivity.

Many of the previously described chromatographic approaches use HILIC due to the high polarity of metformin [16,17,18,19,20,21], which, in our hands, achieved good retention but had interferences and suboptimal peak shape. One previously reported method utilised cation exchange chromatography and achieved good retention [22]. However, the peak width was broad, measuring 24 s at baseline.

We aimed for a robust methodology with high chromatographic resolution power and sharp peaks to optimise sensitivity. Reverse-phase chromatography is the gold standard for these characteristics. However, metformin has little retention on standard reverse-phase columns. As a consequence, using reverse-phase columns results in early elution and reduced chromatographic resolution power [11,23,24,25,26,27,28,29,30,31,32,33,34,35,36]. Hybrid materials applicable to reverse-phase conditions can mitigate the low retention, but also result in relatively broad peaks, with all methods showing peak widths of more than 12 s at baseline [37,38,39,40,41,42,43,44].

Most previously described assays were established for plasma metformin quantification. While broad peak width and low retention are usually not a problem for the bioanalysis of metformin due to its very high plasma concentrations, it is not warranted when developing assays for low metformin concentrations that require more efficient separation of potential interfering substances from the matrix and optimal focusing of metformin during chromatography for maximising sensitivity.

Ion-pairing-based chromatography, such as using the additive TFA, can highly increase the retention of polar compounds and optimise peak shape on reverse-phase columns. This is, however, usually not applicable for mass spectrometry quantification because of the ion suppression caused by the adduct formation with cationic functionalities. In our group, we previously developed a post-column infusion of aqueous ammonia that resolves the adduct formation of TFA with guanidino functions of oligoarginines, allowing for highly sensitive quantification using mass spectrometric detection while concurrently using efficient TFA-based chromatography [14]. However, when using this methodology with TFA on a Waters Acquity BEH C18 Column, we did not obtain enough retention for metformin, as the peak eluted very shortly after the column void volume. Moreover, we had a small shoulder peak, which could be a tautomerisation already described for metformin [36]. We concluded that the ion-pairing with TFA did not result in sufficiently hydrophobic species. Two previously reported methods for metformin quantification made use of TFA/ammonium buffer as a mobile phase additive on a standard reverse-phase column [26] and on a mixed-mode column [12]. Similar to our observation, retention on the standard reverse-phase column was very low. The mixed-mode column achieved better retention, similar to other methods on similar stationary phases. Nevertheless, both methods had peak widths of more than 12 s at baseline.

To improve retention, we therefore replaced the mobile phase additive TFA with a substantially more hydrophobic ion-pairing reagent: HFBA, which has similar ion-pairing capacity due to comparable acidity while having a substantially longer perfluorinated alkane chain (three perfluorinated carbon atoms vs. one in TFA). This led to highly improved strong retention and an excellent peak width of 6 s at baseline that was superior to the previously reported methods for metformin quantification. Our established methodology of using post-column ammonia infusion to avoid ion suppression efficiently resolved the ion-pairing of HFBA and therefore enabled highly sensitive mass spectrometric detection. Due to the very good chromatographic characteristics, the assay demonstrated a high sensitivity that enabled us to easily achieve our desired LLOQ of 0.05 ng/mL (0.38 nM) at a signal-to-noise ratio of around 500, and could potentially support an LLOQ one order of magnitude lower, because the obtained limit of detection (signal-to-noise ratio of 3) was at least 1 pg/mL. Lowering the LLOQ may support interaction studies in cell lines and relevant tissue with lower OCT expression. Further, the facilitation of using reverse-phase chromatography might support the development of simultaneous quantifications of additional substances concurrently with metformin. Additionally, the excellent retention can facilitate the separation of potential interferences and can therefore foster high-sensitivity investigations in other complex matrices.

Because we coupled the HFBA-based LC-MS/MS quantification with a sample processing strategy using a 7% aqueous HFBA solution to conduct cell lysis and protein precipitation concurrently, only one sample transfer step was necessary, and the extracts were directly compatible with the initial conditions of the chromatographic gradient. This resulted in a simple and rapid sample processing method for quantifying metformin in cell pellets. This extraction methodology was very well suited for the hydrophilic metformin, as it produced very clean aqueous extracts with efficient depletion of proteins and lipids, which was underscored by the quantitative extraction recovery and the negligible matrix effect.

After validation, we measured metformin uptake kinetics in the cell lines HEK293-Flp-In-hOCT1, HEK293-hOCT2 and HEK283-hOCT3. To start, we conducted time experiments to evaluate the incubation times needed for optimal experimental settings and to test the intracellular accumulation of metformin in each cell line. We could measure metformin without problems in all cell lines and observed that uptake via OCT2 was faster than that of OCT1 and OCT3. This was in accordance with the literature, where a plateau after 30 s is described for OCT2, whereas OCT1 and 3 did not achieve a plateau even after 10 min (Figure 5a–c) [7,45]. As expected, metformin followed Michaelis–Menten kinetics, and the calculated apparent Km values for metformin transport lay in the range of previously published values obtained in other HEK293 clones with OCT overexpression [45,46,47,48,49].

OCT2 had a much higher efficacy in transporting metformin than the other two OCTs, which is also in accordance with previously published data [8,9]. Even though, in in vitro experiments, it is clear that the in vivo primarily renally expressed OCT2 has a much higher capacity to transport metformin than the hepatic OCT1, which is needed for the effect of metformin [8,9], one should keep in mind that these are artificial systems with a much higher and/or different expression than in hepatic and/or renal tubular cells. Thus, one cannot conclude that the uptake in the liver is lower than the excretion in the kidneys.

To test if our in vitro cell system is suitable for identifying inhibitors of the OCT1-3 transporters, verapamil, a well-known potent inhibitor of OCT1-3 [50], was used. Verapamil inhibited the uptake of metformin in all three tested cell lines with IC_50_ values comparable to previously published ones. OCT1 mediated transport of metformin was inhibited most potently, as previously described in the literature (Figure 6, Table 4) [50].

To conclude, we have established a highly sensitive UPLC-MS/MS method for measuring the intracellular accumulation of the OCT1-3 substrate metformin. This method may allow measuring metformin not only in HEK293-overexpressing cell lines, but also in clinically relevant tissue such as the liver, intestines and kidney cells.

## 4. Materials and Methods

### 4.1. Materials

Cell culture medium DMEM was purchased from PAN Biotech (Aidenbach, Germany), foetal calf serum (FCS) was obtained from Capricorn Scientific GmbH (Ebsdorfergrund, Germany). Supplements, Hank’s buffered salt solution (HBSS), phosphate buffered saline (PBS), CASY® ton, CASY® clean, verapamil, HFBA, metformin hydrochloride and 28% ammonia were obtained from Sigma-Aldrich (Taufkirchen, Germany). Metformin D6 hydrochloride was purchased from Biomol (Hamburg, Germany). Dimethyl sulfoxide (DMSO) and geneticin (G418) were obtained from AppliChem (Darmstadt, Germany). TFA was obtained from Merck (Darmstadt, Germany). Purified water was produced using an arium® mini (Sartorius, Göttingen, Germany) ultrapure water system. The remaining reagents and solvents, methanol (MeOH), acetonitrile (ACN) and formic acid (FA), were purchased from Biosolve (Valkenswaard, The Netherlands) in the highest purity available.

### 4.2. Standard Solutions

Calibration and quality control (QC) stock solutions of metformin were prepared by dissolving two independent weighed samples in 1 mL ultrapure water using volumetric flasks. Further dilutions to achieve the calibration points of 0.05, 0.1, 0.5, 1, 5, 10 and 50 ng/mL, and QC solutions with concentrations of 0.15, 18.75and 37.5 ng/mL were made in water + 0.1% HFBA. The internal standard of metformin D6 was prepared by dissolving 1 mg in 1 mL ultrapure water and was used after further dilutions in water + 0.1% HFBA at a concentration of 1 ng/mL.

### 4.3. Instrumental Analysis Parameters

For testing HILIC chromatography, a UPLC-MS/MS system (Waters, Milford, MA, USA) consisting of a triple-stage quadrupole mass spectrometer (Waters Xevo TQ-XS with Z-spray ionisation and step wave source optimisation) connected to an Acquity classic UPLC® system (Waters Sample Manager, Binary Solvent Manager including cooled sample trays, integrated column heater, degasser and Column Manager) was used. Chromatographic separation was performed using a Waters Cortecs HILIC column (300 Å, 1.7 μm, 2.1 × 50 mm) with an integrated filter disc.

In the final method, quantification was performed on a similar UPLC-MS/MS system (Waters, Milford, MA, USA) consisting of a triple-stage quadrupole mass spectrometer (Waters Xevo TQ-S with Z-spray ionisation and step wave source optimisation) connected to an I-class UPLC® system (Waters Sample Manager, Binary Solvent Manager including cooled sample trays, integrated column heater, degasser and Column Manager). Chromatographic separation was performed on a Waters Acquity BEH C18 Peptide Column (300 Å, 1.7 μm, 2.1 × 150 mm) with an integrated filter disc heated to 60 °C with a flow rate of 0.5 mL/min. The eluent consisted of 0.1% volume (v) aqueous HFBA (aqueous eluent; A) and ACN + 0.1% (v) HFBA (ACN eluent; B). The starting condition of 98% A/2% B was changed after 0.5 min to 63% A/37% B within 2.5 min (0.5 to 3.0 min) and maintained for only 0.1 min before increasing to 5% A/95% B. This was maintained for 0.6 min before being re-equilibrated to the initial ratio. Initial conditions were additionally maintained for 1 min while the Sample Manager prepared the subsequent injection. The total cycle time was 5 min. Post-column addition of 20 µL of 1% aqueous ammonia was performed 2 min after the gradient start in a combined flow state until 3.15 min. For the TFA tests, the same conditions were used; only TFA was used instead of HFBA.

Ionisation parameters of the Z-spray ESI source were manually optimised, and the Xevo TQ-S automatically tuned to the fragments presented in Figure 1 for the substance as well as the IS, using the integrated IntelliStart procedure of the MassLynx V4.2 system software (Waters, Milford, MA, USA). Quantification was performed by multiple reaction monitoring (MRM), using argon collision gas for CID, with MS/MS transitions of the analyte and IS monitored in the positive ion mode. For quantification, the *m/z* transition of 130 → 60 was used for metformin and *m*/*z* 136 → 60 for metformin D6 with a capillary voltage of 0.5 kV; the optimal cone voltage was set to 20 V, and a collision energy of 12 V was used.

### 4.4. Sample Preparation

For the calibration and QC samples, HEK293 cells were used as blank cell pellets. They were dissolved using 100 µL of 7% HFBA in water and were vortexed vigorously before being placed on an ultrasound water bath for 20 min until the cell pellet was completely dissolved. Actual samples were treated similarly. After the addition of the internal standard in each sample (calibration, QCs and actual samples) and 25 µL of water + 0.1% HFBA to the actual samples for volume compensation, samples were vortexed again and then centrifuged at 17,000× *g* for 5 min. Then, 100 µL of the supernatant was transferred to a 96-well collection plate (800 µL; Waters, Milford, MA, USA), and 20 µL was injected into the UPLC-MS/MS system.

### 4.5. Validation

For the validation of the method, applicable requirements from the FDA and EMA guidelines for the validation of bioanalytical methods were considered [51,52]. Three validation batches containing blanks and all calibration points in duplicates and sextuplets of LLOQ, QC A, QC B and QC C as well as system suitability tests were performed. Matrix effect and recovery were measured in triplicates using the spiking of samples after processing and eluents [53], as well as stability samples for two and four weeks at −20 °C and freeze thaw samples. The last samples were thawed three times for 3 h and frozen again for at least 24 h until the third final thawing step immediately before measurement. To test the stability of the prepared samples in the 96-well samples in case of delay in measurement, autosampler stability was tested for 24 h at 10–15 °C. For testing benchtop stability, blanks were spiked with 25 µL of the respective QC solution. Samples were vortexed briefly and left on the benchtop for 4 h before processing.

### 4.6. Cell Lines Used and Their Culture

The HEK-hOCT2 and HEK-hOCT3 cell lines were a kind gift from Herrmann Koepsell and have been generated and characterised previously [54]. HEK293 cells (available at ATCC, Manassas, VA, USA), HEK293-OCT2 and HEK293-OCT3, were cultured under standard cell culture conditions in DMEM with 10 % FCS, 2 mM glutamine, 100 U penicillin/100 μg streptomycin and 600 µg/mL geneticin for the OCT-overexpressing cell lines. The stably hOCT1-overexpressing HEK293-Flp-In-hOCT1 cells have been generated and characterised previously [55,56] and were cultured under standard cell culture conditions in DMEM with pyruvate, with 10% FCS, 2 mM glutamine and 100 U penicillin/100 μg streptomycin sulphate.

### 4.7. Establishment of OCT-Inhibition Assays in HEK293 Cell Lines Overexpressing OCT1, OCT2, or OCT3

Before establishing an OCT inhibition assay, we characterised the metformin uptake kinetics in OCT1-, OCT2- or OCT3-overexpressing cells. For assessing the time-dependent uptake of metformin, cells were detached, washed once and the cell volume was measured in a CASY^®^ cell counter (OMNI Life Science GmbH + Co KG, Bremen, Germany). For each incubation, 1 × 10^6^ cells of the respective cell line were resuspended and pre-incubated in low-binding 1.5 mL tubes for 5 min in 150 µL HHBSS at 37 °C on a rotary shaker. After adding 150 µL of 20 µM metformin solution for OCT1 and OCT3 and 4 µM for OCT2 (in HHBSS, final concentration: 10 µM or 2 µM respectively), cells were incubated from 0.5 up to 30 min on a rotary shaker at 37 °C. After pelleting at 4 °C, cells were washed twice with ice-cold HHBSS, and the pellet was frozen at –20 °C until extraction, which always took place within one week (cf. 4.4). For investigating the substrate-dependent kinetics of metformin, the uptake was conducted as described above using 0.1 µM–10 mM metformin and incubation times of 10 min for OCT1 and 5 min for OCT2 and OCT3.

For measuring the inhibition of metformin uptake in the OCT-overexpressing cell lines, the assay was conducted as described above, using a final metformin concentration of 10 µM and a preincubation time of 20 min, with the inhibitor verapamil (final concentration 0.01–500 µM for OCT1 and 0.3–300 µM for OCT2 and OCT3) and 2 min (OCT2) or 10 min (OCT1 and OCT3) incubation with metformin.

All experiments were conducted with technical triplicates and at least three biological triplicates.

### 4.8. Calculations and Statistical Methods

Metformin and metformin D6 structures were drawn using PubChem Sketcher V2.4. For the validation calculations, custom sheets were created using Microsoft Excel, and all other figures were made using GraphPad Prism version 10 unless stated otherwise. For the uptakes, a four-parameter regression fit was used. Calibration curves were calculated with weighted linear regressions (1/x^2^) using TargetLynx software (V4.2 Waters), which was also used for the chromatograms and spectra.

## 5. Conclusions

In conclusion, we have established a highly sensitive UPLC-MS/MS method to quantify the uptake of metformin in cells and validated it according to the FDA and EMA requirements. Using this method, we have established an in vitro model that can be used to quantify metformin uptake in cells and identify OCT1-3 inhibitors, and thereby also proved the suitability of the UPLC-MS/MS method for the quantification of very low metformin amounts in cells. This may also support interaction studies in cell lines and relevant tissues with low OCT expression. Further, the method presented supports the development of simultaneous quantifications of additional compounds concurrently with metformin and high-sensitivity investigations in other complex matrices.

## Figures and Tables

**Table 1:** Summary of QC results in cell pellets for metformin.

	LLOQ 0.05 ng/mL	QC A 0.15 ng/mL	QC B 18.7 ng/mL	QC C 37.5 ng/mL
Intra-day				
1 Mean [ng/mL]	0.0536	0.141	16.7	32.0
Accuracy (%)	107	93.9	89.0	85.4
Precision (% CV)	9.97	3.49	3.09	3.85
2 Mean [ng/mL]	0.0471	0.132	16.8	34.0
Accuracy (%)	94.2	88.3	89.4	90.7
Precision (% CV)	9.60	3.04	3.24	4.11
3 Mean [ng/mL])	0.0498	0.142	16.8	33.3
Accuracy (%)	99.7	95.0	89.6	88.9
Precision (% CV)	8.28	6.47	5.36	2.94
Inter-day				
Mean [ng/mL]	0.0512	0.139	16.8	33.3
Accuracy (%)	102	92.7	89.4	88.9
Precision (% CV)	11.2	5.40	3.87	3.22

n = 6; CV—coefficient of variation; LLOQ—lower limit of quantification; QC—quality control.

**Table 2:** Summary of stability results in cell pellets for metformin.

	QC A 0.15 ng/mL	QC B 18.7 ng/mL	QC C 37.5 ng/mL
Autosampler (24 h at 10–15 °C)			
Accuracy (%)	100	102	103
Bench-top (4 h) Accuracy (%)	101	90.4	87.5
Freeze and thaw			
Accuracy (%)	92.7	99.7	96.7
Four weeks at −20 °C			
Accuracy (%)	97.3	95.8	86.4

QC—quality control; n = 3.

**Table 3:** Summary of matrix effect and recovery.

	QC A 0.15 ng/mL	QC B 18.7 ng/mL	QC C 37.5 ng/mL
Matrix effect (%)	114	95.7	96.9
IS-normalised matrix effect (%)	115	94.5	98.7
Recovery (%)	94.2	100	96.6

QC—quality control; n = 3.

**Table 4:** Kinetic parameters of metformin transport in HEK293-Flp-In-hOCT1, HEK293-hOCT2 and HEK293-hOCT3 cells.

Kinetic Parameter	OCT1	OCT2	OCT3
Km [mM]	24.4 ± 10.1	6.3 ± 0.9	4.6 ± 1.1
V_max_ [amol/min/cell]	515 ± 233	1513 ± 706	1197 ± 318
IC_50_ of metformin transport inhibition by verapamil [µM]	4.6 ± 1.4	23.3 ± 6.3	28.4 ± 3.0

Data represent mean ± S.D. for n = 3.

## Data Availability

Data are made available upon reasonable request.
